# Simultaneous FTIR and Raman Spectroscopy in Endometrial Atypical Hyperplasia and Cancer

**DOI:** 10.3390/ijms21144828

**Published:** 2020-07-08

**Authors:** Edyta Barnas, Joanna Skret-Magierlo, Andrzej Skret, Ewa Kaznowska, Joanna Depciuch, Kamil Szmuc, Kornelia Łach, Izabela Krawczyk-Marć, Jozef Cebulski

**Affiliations:** 1 Institute of Health Sciences, College of Medical Sciences, University of Rzeszow, Kopisto 2a, 35-959 Rzeszów, Poland; ebarnas@interia.eu; 2 Medical College of Rzeszow University, Institute of Medical Sciences, Kopisto 2a, 35-959 Rzeszów, Poland; joannaskret@wp.pl (J.S.-M.); magierlo@interia.pl (A.S.); 3 Department of Pathomorphology, Chair of Morphological Sciences, College of Medical Sciences, University of Rzeszow, Kopisto 2a, 35-959 Rzeszow, Poland; e.kaznowska@op.pl; 4 Institute of Nuclear Physics, Polish Academy of Science, Radzikowskiego 152, 31-342 Krakow, Poland; 5 Institute of Physics, College of Natural Sciences, University of Rzeszow, - St. Pigonia 1, 35-310 Rzeszów, Poland; szmuc.kamil@gmail.com (K.S.); cebulski@ur.edu.pl (J.C.); 6 Clinic of Paediatric Oncology and Haematology, College of Medical Sciences, University of Rzeszow, Kopisto 2a, 35-959 Rzeszow, Poland; kornelia_lach@wp.pl; 7 Department of Human Histology, Chair of Morphological Sciences, College of Medical Sciences, University of Rzeszow, Kopisto 2a, 35-959 Rzeszow, Poland; isabelkrawczyk@interia.pl

**Keywords:** endometrial cancer, atypical hyperplasia, FTIR spectroscopy, Raman spectroscopy, correlation test

## Abstract

Currently, endometrial carcinoma (EC) is the most common genital cancer in high-income countries. Some types of endometrial hyperplasia (EH) may be progressing to this malignancy. The diagnosis of EC and EH is based on time consuming histopathology evaluation, which is subjective and causes discrepancies in reassessment. Therefore, there is a need to create methods of objective evaluation allowing the diagnosis of early changes. The study aimed to simultaneously asses Fourier Transform Infrared (FTIR) and Raman spectroscopy combined with multidimensional analysis to identify the tissues of endometrial cancer, atypical hyperplasia and the normal control group, and differentiate them. The results of FTIR and Raman spectroscopy revealed quantitative and qualitative changes in the nucleic acid and protein in the groups of cancer and atypical hyperplasia, in comparison with the control group. Changes in the lipid region were also observed in Raman spectra. Pearson correlation coefficient demonstrated a statistically significant correlation between Raman spectra for the cancer and atypical hyperplasia groups (0.747, *p* < 0.05) and for atypical hyperplasia and the controls (0.507, *p* < 0.05), while FTIR spectra demonstrated a statistically significant positive correlation for the same group as in Raman data and for the control and cancer groups (0.966, *p* < 0.05). To summarize, the method of spectroscopy enables differentiation of atypical hyperplasia and endometrial cancer tissues from the physiological endometrial tissue.

## 1. Introduction

The endometrium lining of the uterine cavity is a structure subjected to changes in the menstrual cycle or pregnancy, as well as during such periods of a woman’s life as puberty and menopause. Currently, endometrium becomes the subject of scientific interest due to the growing incidence of endometrial cancer in developed countries [1].

Endometrial hyperplasia is of particular interest since certain types of this hyperplasia may predispose to endometrial cancer. In developed countries, there are an estimated 200,000 new cases of endometrial hyperplasia per annum [2].

Simple and complex hyperplasia without atypia are not considered risk factors for endometrial cancer, whereas forms of hyperplasia with cytological atypia pose such a risk. The cases of endometrial cancer include type I cancer preceded by estrogen-stimulated hyperplasia and type II with a different mechanism of formation [3,4]. 

Prevalence of endometrial hyperplasia (EH) is approximately three times higher than endometrial cancer (EC) and atypical EH may be progressing to EC lesions [5]. Moreover, in the retrospective analysis of 170 patients with EH, subjected uterine curettage observed progression to EC in 13 patients during a 13.4-year follow-up. The progression from EH to EC amounted to 1% in the case of simple hyperplasia (SH, without atypia), 3% for complex hyperplasia (CH, without atypia), 8% for simple atypical hyperplasia (SAH) and 29% for complex atypical hyperplasia (CAH), respectively [6]. The distinction of these four types of hyperplasia is based on microscopic assessment largely based on quantitative, subjective evaluation.

Lacey et al. analyzed 138 cases of EH which progressed to EC for at least 1 year following EH diagnosis. The risk of developing EC after EH diagnosis (including simple and complex EH types) was estimated at 40%. However, in the case where the atypia was absent, the risk of progression was only at 10%. The authors postulated the need to improve the sensitivity and specificity of methods of EH diagnosis with particular focus on the rare non-atypical EH lesions likely to progress to EC [7].

Other studies based on the re-evaluation of preparations diagnosed with atypical hyperplasia showed that in these cases both diagnosed and undiagnosed cases were found [8,9,10]. Hence, methods based on research other than microscopy are sought. 

Raman and infrared (IR) spectroscopy are the methods, which provide detailed information about chemical composition of different matters including biological samples. These techniques allow the determination of vibrations of functional groups, which build carbohydrate, deoxyribonucleic acid (DNA), protein and lipid compounds [11]. Moreover, the physical fundamentals of Raman and IR spectroscopy are different, therefore these methods are complementary [12]. 

Many papers have reported on using vibrational spectroscopy methods to determine chemical changes in the breast [13], lung [14], bone [15], thyroid [16] and brain [17] under the carcinogenesis. Moreover, spectroscopy data are based on physical models, which can be used in monitoring cancer treatment processes. Furthermore, IR spectroscopy enables the detection of changes between the control and cancer ovarian tissues [18]. There are several studies in the literature where IR spectroscopy was used to analyze endometrial tissues. Researchers studied normal endometrial tissue using two spectroscopic techniques (synchrotron radiation-based Fourier- transform infrared (SR-FTIR) microspectroscopy and globar focal plane array-based FTIR spectroscopy), to determine the putative stem cells which may represent clonogenic cells important in carcinogenesis. They identified a location of putative stem cells indicating PO_2_ in DNA and RNA nucleic acids and amide I and II vibrations as major discriminating factors [19]. Endometriosis is endometrial pathology defined as the spread of endometrial tissue outside the endometrial cavity. Like malignant lesions, endometriosis demonstrates atypia and is able to adhere, invade and disseminate [20]. The endometriosis may be diagnosed with a laparoscopy; therefore, research focuses on new non-invasive methods. So far, only one study presents the application of Raman spectroscopy among women suffering from endometriosis [21].

To the best our knowledge, the literature presents only three studies where authors analyzed pathological endometrial tissues using FTIR techniques [22,23,24], but Raman spectroscopy has not been studied so far in this respect. In this study, Raman and FTIR spectroscopy were applied for the first time in multidimensional analysis to identify and differentiate atypical hyperplasia, endometrial cancer tissues and the physiological endometrial tissue.

## 2. Results

The spectrum analysis performed in this study involved specifying individual peaks in the Raman and FTIR spectra of three groups: physiological endometrial tissue, atypical hyperplasia and cancer samples.

**Figure 1:**
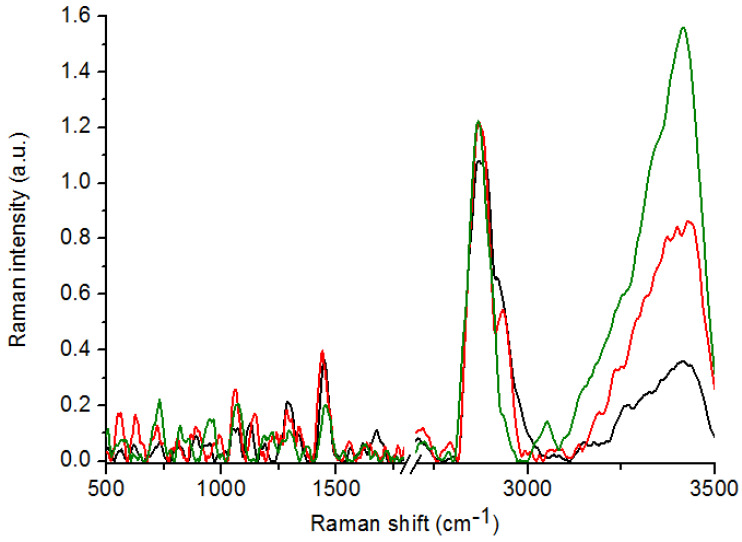
Raman spectra of: control (*n* = 17, green spectrum); atypical hyperplasia (*n* = 12, black spectrum) and cancer (*n* = 16, red spectrum) tissues.

In the Raman spectra of atypical hyperplasia and cancer tissues, absence of peaks originating from stretching vibrations of CO, CC and OCH from rings of polysaccharides and pectin, were noticed in comparison with the control samples (Figure 1). Moreover, in the Raman spectrum of atypical hyperplasia tissues, lack of peaks corresponding to Proline, hydroxyproline, tyrosine, PO^2−^ stretching from nucleic acids, Tryptophan and C–O stretching from lipids, as well as CH_2_ stretching from lipids, was observed in comparison with the control sample. A significant shift of peaks originating from the amide I and CH_3_ groups from lipids was visible when compared to the Raman spectra of the atypical hyperplasia and control tissues. Furthermore, in the cancer tissues, a significant shift of peaks corresponding to the tryptophan, amide I and CH_2_ groups from lipids, was observed in comparison with the control samples. When comparing the Raman spectra of atypical hyperplasia and cancer samples, in the first one, peaks corresponding to the proline, hydroxyproline, tyrosine and PO^2−^ stretching groups from nucleic acids (~815 cm^−1^), tryptophan (~1370 cm^−1^) and the C–O and CH_2_ vibrations from lipids (~1740 cm^−1^ and 2919 cm^−1^, respectively), were not observed. Moreover, a significant shift of peaks originating from C–C stretching from proline and hydroxyproline, C–C, tryptophan (protein assignment) and amide I, were noticed in the Raman spectrum of atypical hyperplasia in comparison with the cancer spectrum. Furthermore, in the Raman spectrum of atypical hyperplasia, a lower Raman intensity in the range between 500 and 1800 cm^−1^ and a higher intensity in the CH_2_ lipids region (~2800 cm^−1^), were visible. A description of the vibrations which were visible in the Raman spectra with the differences between analyzed samples is presented in Table 1.

FTIR spectra can be divided into three basic groups corresponding to the basic building blocks of cell components. The following spectral regions can be distinguished: of the region 1000–1250 cm^−1^ responsible for the nucleic acid strand, the region 1500–1560 cm^−1^, 1600–1700 cm^−1^ of protein and the last region of lipids from a 2800–3000 cm^−1^ wavenumber.

**Figure 2:**
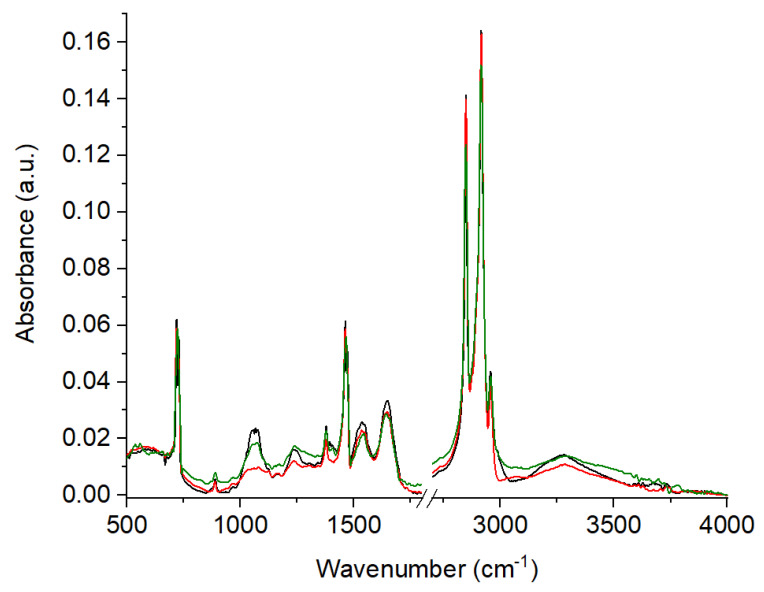
Fourier Transform Infrared (FTIR) spectra of: control (*n* = 17, green spectrum); atypical hyperplasia (*n* = 12, black spectrum) and cancer (*n* = 16, red spectrum) tissues.

In the FTIR spectra of the control and atypical hyperplasia samples, the absence of peaks originating from the stretching vibrations of C–O carbohydrates (1124 cm^−1^) and deformation N–H cytosine (1304 cm^−1^) were noticed in comparison with the carcinoma samples (Figure 2, Table 2). In the spectrum of carcinoma, the lack of peaks corresponding to CH_2_ wagging for proline from amino acids and collagen (~1341 cm^−1^) and carbonate band ʋ_2_(CO_3_^2−^) of a wavenumber of ~1407 cm^−1^ was observed. Of note is the peak found at the level of 1073 cm^−1^, corresponding to the C–O stretching mode of the C–OH groups of serine, threonine and tyrosine of protein, shifted towards a lower wavenumber at 7 cm^−1^ and towards a higher wavenumber at 7 cm^−1^ for atypical hyperplasia and carcinoma, respectively. The characteristic absorption band belonging to the stretching vibrations of C–O, C–C and C–O–H deformation originated from proteins, glycogen and carbohydrates was found at the level of 1171 cm^−1^. The peak in the region of 1240 cm^−1^ arose from N–H bending, C–C and C–N stretching vibrations of amide III. Interestingly, these last two peaks shifted 4 and 3 cm^−1^, respectively, towards lower wavenumbers for the carcinoma samples alone.

The next maximum, located at 1407 cm^−1^ was attributed to carbonate band stretching and shifted towards a lower wavenumber at 13 cm^−1^ for atypical hyperplasia alone. The peaks of the wavenumber at 1466 and 1544 cm^−1^ corresponding to the CH_2_ scissoring modes and amide II shifted towards lower wavenumbers at 4 and 9 cm^−1^, respectively, for both the hyperplasia and carcinoma patients. Amide I, one of the main protein conformers, had absorption noticed in the area of 1642 cm^−1^. The higher wavenumbers (7 cm^−1^ for atypical hyperplasia and 4 cm^−1^ for carcinoma) were observed for both pathological tissues. 

Changes were also found in the peak placement between the atypical hyperplasia and carcinoma tissues, mainly corresponding to proteins conformations. Analysis of the FTIR spectrum showed the massive shift (14 cm^−1^) of the C–O stretching mode of the C–OH groups of serine, threonine, and tyrosine for carcinoma (1080 cm^−1^) in comparison to hyperplasia (1066 cm^−1^). In the case of amide I and amide III compounds, the shift was found towards a lower wavenumber at 3 and 4 cm^−1^, respectively. 

A summary of the peaks from FTIR spectra with the differences between analyzed samples is presented in Table 2.

**Figure 3:**
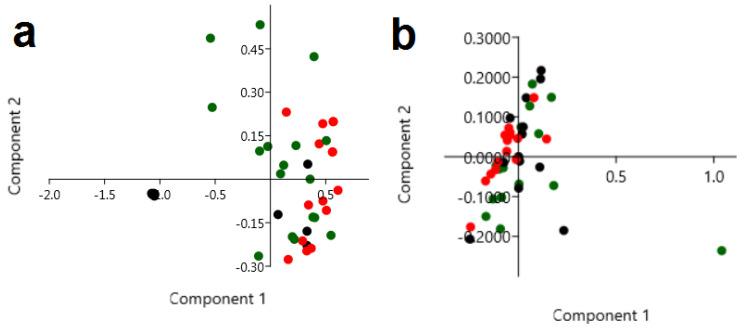

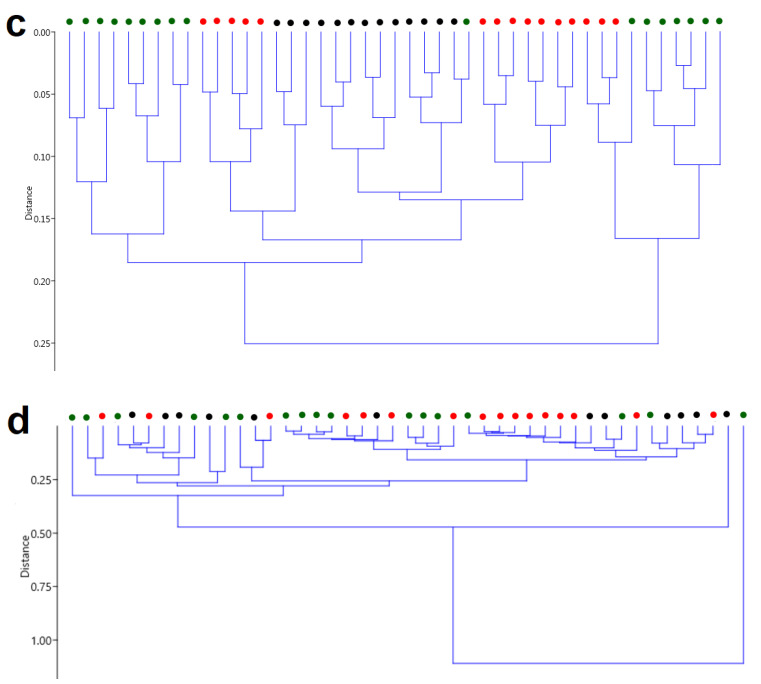
Principal component analysis (PCA) and hierarchical cluster analysis (HCA) analysis of Raman (**a**,**c**) and FTIR (**b**,**d**) spectra of control (*n* = 17, green dot); atypical hyperplasia (*n* = 12, black dot) and cancer (*n* = 16, red dot) tissues. Two-dimensional (2D) scores plot of endometrium tissues samples, due to differences in chemical compositions presented through the Raman spectral regions between 800–1800 cm^−1^.

To resolve whether it was sufficient to detect only the differences between the studied samples, principal component analysis (PCA) was used to analyze the obtained Raman (Figure 3a) and FTIR (Figure 3b) spectra. PCA analysis obtained from the Raman data showed that most of the control samples were separated from the Raman data obtained for the cancer and atypical hyperplasia tissues. Moreover, four out of thirteen Raman spectra of atypical hyperplasia were in the same part of the PCA plot as the Raman spectra of cancer. This means that it is possible to distinguish the Raman spectra of the control and atypical hyperplasia, as well as cancer tissues and atypical hyperplasia. While PCA analysis obtained for FTIR data (Figure 3b) showed that only the spectra of cancer tissues were similar to each other and could be separated from the other analyzed samples, PCA analysis also showed that, using FTIR spectroscopy, it was not possible to distinguish the spectra of the atypical hyperplasia and control tissues. Furthermore, hierarchical cluster analysis (HCA) showed that almost all Raman spectra (Figure 3) of atypical hyperplasia (black dot) created one separate group of similarity, when the control samples created two groups, however, these groups were not similar to atypical hyperplasia and cancer tissues. Moreover, HCA analysis showed a higher similarity between atypical hyperplasia and cancer tissues than between the control and atypical hyperplasia or the control and cancer. In the case of the FTIR results (Figure 3d) HCA analysis showed a similarity only between almost all cancer tissues; however, the control samples or atypical hyperplasia samples did not create homogenous groups.

The Pearson correlation coefficient presented in Table 3 showed that it possible to determine the correlation between the character/shape of the Raman and FTIR spectra obtained for the control, atypical hyperplasia and cancer tissues. The data can be summarized as follows. The Raman spectra demonstrated a positive and significant correlation between the nature of the spectrum of atypical hyperplasia and cancer tissues, as well as between the atypical hyperplasia and control tissues. However, the Pearson correlation coefficient showed no significant positive correlation between the control and cancer tissues. The Pearson correlation coefficient for FTIR data indicated that there was also a positive correlation between the FTIR spectra of atypical hyperplasia and cancer, as well as between the atypical hyperplasia and control tissues. Furthermore, the Pearson correlation coefficient also showed that there was a significant correlation between the FTIR spectra of the cancer and control tissues. These results are also visible in the PCA and HCA analysis obtained for Raman and FTIR spectra.

## 3. Discussion 

In our innovative study both Raman and FTIR spectroscopy methods were used because they analyze the vibrational modes of specific functional groups qualitatively and quantitatively. The oldest study showed differences among endometrial cancer and normal tissues using FTIR in 17 females subjected to total abdominal hysterectomy (13 patients with grade I endometrial adenocarcinoma and 4 with grade III adenocarcinoma). The spectra of the malignant samples revealed the symmetric and asymmetric stretching bands of the phosphodiester backbones of nucleic acids, the CH stretching region, the C–O stretching bands of the C–OH groups of carbohydrates and cellular protein residuals and the pressure dependence of the CH_2_ stretching mode [22]. These results were in line with our results, but it is worth noting that the authors used the outdated scale. Our study used the newer histopathological classification system by The World Health Organisation (WHO) established in 1994 with a revision in 2003. It includes four categories of endometrial hyperplasia and is widely used within current clinical gynecological practice. In our study, the latest criteria for endometrial intraepithelial neoplasia (EIN) were not used. The EIN concept incorporated morphometric and molecular diagnostics to differentiate hyperplastic endometrial lesions into two groups: (a) benign EH and (b) EIN [38].

Researchers used transmission FTIR microspectroscopy to distinguish non-tamoxifen associated from tamoxifen-associated endometrial samples. The differences were observed in the protein region for benign or endometroid carcinoma tissues (1800–1480 cm^−1^) [23]. Differences inmethodology prevent us, however, from comparing our results with this study.

Moreover, evaluated different endometrial tissues from 76 patients undergoing hysterectomy, including 36 of endometrial cancer. AT-FTIR was used in this preliminary study allowing the differentiation of benign and malignant endometrial tissues (including various subtypes of both). The differences were presented in the structural protein (amide I/II region), these were larger in endometroid than non-endometroid cancers, especially in Amide I [24]. In our study, we also observed the changes in peak placement between atypical hyperplasia and carcinoma tissues, mainly corresponding to protein conformations. Analysis of the FTIR spectrum showed the massive shift (14 cm^−1^) of the C–O stretching mode of the C–OH groups of serine, threonine, and tyrosine for carcinoma (1080 cm^−1^) in comparison to hyperplasia (1066 cm^−1^). For amide I and amide III compounds, we noticed the shift towards a lower wavenumber at 3 and 4 cm^−1^, respectively, while in the Raman spectra of atypical hyperplasia and cancer tissues, the absence of peaks originating from stretching vibrations of CO, CC and OCH from the ring of polysaccharides and pectin, was noticed in comparison with the control samples. Furthermore, in the cancer tissues, a significant shift of peaks corresponding to the tryptophan, amide I, and CH_2_ groups from lipids was observed in comparison with the control samples. In addition, PCA analysis obtained from the Raman data showed that most of the control samples were separated from the Raman data obtained for cancer and atypical hyperplasia tissues, while PCA analysis obtained for FTIR data showed that only the spectra of cancer tissues are similar to each other and can be separated from the other analyzed samples. It is not possible to distinguish the spectra of the atypical hyperplasia and control tissues. HCA analysis showed that almost all Raman spectra (Figure 3b) have a higher similarity between atypical hyperplasia and cancer tissues, than between the control and atypical hyperplasia or the control and cancer. In the case of the FTIR results (Figure 3d), HCA analysis showed a similarity only between almost all cancer tissues, however, the control samples or atypical hyperplasia samples did not create groups of similarity. 

Based on the analyses, it can be concluded that both FTIR and Raman spectroscopy provide qualitative and quantitative analyses of the vibrational modes of specific functional groups. Therefore, they are sometimes referred to as sister or complementary techniques. However, principal component analysis, as well as hierarchical component analysis (Figure 3), showed that, using only Raman spectroscopy, it is possible to distinguish the control samples from the atypical hyperplasia and cancer tissues. Moreover, the Pearson correlation coefficient obtained from the Raman results showed that the chemical compositions of atypical hyperplasia and cancer tissues were more correlated than the chemical composition characteristics for atypical hyperplasia and the control as well as the control and cancer tissues. However, in the case of the FTIR results, the Pearson correlation coefficient showed that the correlation between chemical compositions of the control and cancer tissues was more significant than for the tissues with atypical hyperplasia and cancer features. It may result from the fact that both methods are based on different physical fundamentals: change of the dipole moment in the case of FTIR, whilst change of bond polarity in the case of Raman spectroscopy [39,40]. Consequently, in the FTIR and Raman spectra a difference in the intensity of functional groups, as well as their absence, can be observed. However, in the literature information can be found about the risk of cancer disease in women with atypical hyperplasia, as well as about the similarity of the microscopy images of ovaries with cancer cells and atypical hyperplasia [41,42]. This could suggest that Raman spectroscopy offers more accurate results to distinguish the ovarian tissues with analyzed diseases. 

During carcinogenesis in endometrium from simple hyperplasia through hyperplasia with atypia to cancer, genomic changes occur (such as aberrations in sub-regions: dup 14q32.33, del15q11.2, assessed by array Comparative Genomic Hybridization) [43]. In parallel, changes in the proteome assessed by mass spectrometry are also found [44]. 

No unequivocal endometrial cancer biomarker was found in the above studies. Our FTIR and Raman spectroscopy study may be a novel step in the search for this marker.

## 4. Materials and Methods

The study was approved by the institutional Bioethics Committee at the University of Rzeszow (Resolution No. 1/05/2019, dated 9 May 2019). A cross-sectional study was conducted from July 2019 to December 2019. The study was conducted on tissue samples from 45 patients. Among them, 16 suffered from endometrial cancer, 12 from atypical hyperplasia and 17 constitute the normal control group. The control group consisted of 17 patients with an average age of 62 years. Sixteen patients with an average age of 61 years with endometrial cancer were included in this study. The last group of 12 patients with an average age of 55 years were patients with the diagnosis of atypical hyperplasia. The tissue material came from a patient from the Podkarpackie Gynecology and Obstetrics Ward Oncology Centers in Brzozow, KSW Gynecology and Obstetrics Clinic No. 1 in Rzeszow and the Municipal Hospital in Rzeszow.

### 4.1. Materials Preparation

The samples collected from the women suffering from cancer, atypical hyperplasia and without pathological lesions were prepared for a histopathology evaluation in the uniform manner. The tissue samples were initially incubated in a liquid fixative for about 12 h. Next, ethanol was gradually replaced with xylene with a 10% increasing step, (from 50% to 99.8%). A paraffin infiltration of the tissue was performed at the temperature of 52 °C. Tissue blocks were embedded in paraffin by inserting the tissue section with the appropriate spatial orientation into a metal mold and pouring in molten wax. Each section was flattened on a hot water surface. For the spectroscopic measurements, the obtained samples were placed on CaF_2_ slides. The size of the samples used for the spectroscopic measurements was about 5 mm and the thickness 10 µm. Moreover, for each obtained sample, an immunohistochemical diagnostic was performed to confirm the presence or absence of cancer cells.

### 4.2. FT-Raman Measurement

An FT-Raman spectra measurement was performed using a Nicolet NXR 9650 FT-Raman Spectrometer (Thermo Fisher Scientific, Waltham, MA, USA) with Nd: YAG laser source (1064 nm) and a Germanium detector. A spectral range of 150 to 3700 cm^−1^ and a laser power of 500 mW were used. This power was constant for each sample and each measurement. An unfocused laser beam was used of approximately 100 μm in diameter and a spectral resolution of 8 cm^−1^. Raman spectra were processed using the Omnic/Thermo Scientific software (Thermo Fisher Scientific) based on 64 scans for measurements, which means the number of scans performed on one spot was 64, and the obtained spectrum was an average of these scans. All spectra were smoothed using the Savitzky–Golay algorithm for 7 points. Moreover, all spectra were normalized using vector normalization. Additionally, baseline corrections using a Rubberband correction were done. All these operations were performed using OPUS software (Bruker Optik GmbH, Ettlingen, Germany). Moreover, each sample was measured three times in three different places. Next, the spectra were averaged and this average was used for statistical analysis.

### 4.3. FTIR Measurements

The Fourier transform infrared spectrometer (FTIR), Vertex 70v from Bruker (Bruker, Ettlingen, Germeny), was used for the measurements. The samples were examined in the mid-IR range using attenuated total reflection (ATR) with diamond crystal. Data points were collected at a resolution of 2 cm^−1^ (32 scans). The analysis of the 800–3500 cm^−1^ wavenumber spectral range was performed. All measurements were made in triplicate and the next average spectrum was obtained for each sample. Data were processed using OPUS 7.0 Bruker Optik GmbH 2011. Vector normalization was used to normalize the spectra. A Rubberband baseline correction was also performed.

### 4.4. Multivariate Analysis and Pearson Correlation Coefficient

Principal component analysis (PCA) was performed to obtain information about the spectra variation among the sample types. PCA allowed dimensionality reduction reducing the number of variables for dataset, while maintaining as much variance as possible. Moreover, to obtain information about the similarity between the samples, hierarchical cluster analysis (HCA) was performed with Euclidean distance and using the paired group (UPGMA) algorithm. Hierarchical cluster analysis is a popular method for cluster analysis in big data research and data mining aiming to establish a hierarchy of clusters. As such, HCA attempts to group subjects with similar features into clusters. These two procedures were performed based on the selected spectral regions between 800 and 1800 cm^−1^. For this purpose, 518 points from the FTIR spectra and 259 points from the Raman spectra were analyzed. PCA as well as HCA analyses were performed for all obtained spectra, as well as for the average of spectra from each sample type. The correlation between the Raman and FTIR spectra in the control, atypical hyperplasia, and cancer groups was investigated with the Pearson correlation coefficient, adopting the level of statistical significance at *p* < 0.05 and a 95% confidence interval. For this purpose, fingerprint regions of the FTIR and FT-Raman spectra were used. All analyses were performed with Past 3.0 software (Øyvind Hammer, Natural History Museum, University of Oslo).

## 5. Conclusions

To the best of our knowledge, this study is the first to use both Fourier Transform Infrared and Raman spectroscopy combined with multidimensional analysis to identify and differentiate tissues of endometrial cancer, atypical hyperplasia and the normal control group.

The obtained results showed, that in comparison with the control group, the most visible structural changes in the non-control groups, which were observed as shift or absence of peaks, were noticed in the Raman and FTIR regions originating from DNA, RNA and proteins. The structural changes in the lipids were also visible in the Raman spectra. Consequently, the FTIR spectra showed a very significant decrease in the maximum absorbance corresponding to nucleic acid. Interestingly, this decrease intensified with the lesion from atypical hyperplasia to cancer. Moreover, a multivariate analysis and the Pearson correlation coefficient indicated that Raman spectroscopy allowed better differentiation between the control tissues and the atypical hyperplasia and cancer tissues, which may be related to the tissue structure and symmetry of the functional groups creating the tissue. However, our results may suggest the possibility of using the change in the individual peaks in the FTIR and Raman spectra as a potential means of the differentiation of cancer and atypical hyperplasia from normal endometrial tissues samples.

Further studies are needed to properly evaluate the role of Raman and FTIR spectroscopy as a diagnostic tool in carcinogenesis.

## Figures and Tables

**Table 1:** Raman shift with corresponding vibrations described in the Raman spectra of control (*n* = 17), atypical hyperplasia (*n* = 12) and cancer (*n* = 16) tissues [25,26,27,28,29].

No.	Control (I)	Athypical Hyperplasia (II)	Carcinoma (III)	ΔI-II [cm^−1^]	ΔI-III [cm^−1^]	ΔII-III [cm^−1^]	Vibrations
1	812	*	818	max	−6	max	Proline, hydroxyproline, tyrosine, PO^2−^ stretching from nucleic acids
2	890	884	880	6	10	4	C-C stretching from proline and hydroxyproline
3	1011	*	*	max	max	0	Stretching vibrations of CO, CC, OCH from ring of polysaccharides and pectin
4	1065	1064	1066	1	−1	−2	PO^2−^ stretching from nucleic acids
5	1293	1296	1299	−3	−6	−3	Phosphodiester groups in nucleic acids
6	1359	*	1374	max	−15	max	Tryptophan
7	1447	1447	1447	0	0	0	CH_2_ bending from lipids and proteins
8	1553	1550	1558	3	−5	-8	C=C, tryptophan (protein assignment)
9	1695	1675	1668	20	27	7	Amide I
10	1776	*	1723	max	53	max	C=O stretching from lipids
11	2798	2720	2758	78	40	−38	CH_3_ stretching from lipids
12	*	2869	2871	max	max	−2	CH_2_ stretching from lipids

*-Band not visible in the spectrum; max–maximum differences between spectra caused by absence of vibrations in one of the analyzed spectra; ΔI-II, ΔI-III, ΔII-III: differences in the peak positions between analyzed samples.

**Table 2:** Fourier Transform InfraRed (FTIR) shift with corresponding vibrations described in the FTIR spectra of control (*n* = 17), atypical hyperplasia (*n* = 12) and cancer (*n* = 16) tissues [23,24,30,31,32,33,34,35,36,37].

No.	Control (I)	Athypical Hyperplasia (II)	Carcinoma (III)	ΔI-II [cm^−1^]	ΔI-III [cm^−1^]	ΔII-III [cm^−1^]	Vibrations
1	889	889	889	0	0	0	C-C, C-O deoxyribose, fatty acid, saccharide
2	1073	1066	1080	7	−7	−14	C-O stretching mode of C-OH groups of serine, threonine, and tyrosine of protein
3	*	*	1124	max	max	max	νC-O Carbohydrates
4	1171	1169	1167	2	4	2	ν(C-O), ν(C-C), def. C-O-H (proteins, glycogen, carbohydrates)
5	1240	1241	1237	−1	3	4	Amide III (N-H bending, C-N stretch, C-C stretch) (proteins, DNA, phospholipids)
6	*	*	1304	max	max	max	Deformation N-H cytosine
7	1341	1340	*	1	max	max	CH_2_ wagging for proline (amino acids and collagen)
8	1377	1378	1378	−1	−1	0	CH_3_, CH_2_ wagging (lipids/proteins)
9	1407	1394	*	13	max	max	carbonate band ʋ(CO_3_^2−^)
10	1466	1462	1462	4	4	0	CH_2_ group scissoring modes
11	1544	1535	1535	9	9	0	Amide II due to N-H bending and C-N stretching of proteins
12	1642	1649	1646	−7	−4	3	Amide I (ν(C=O), ν(CN), γ(CCN), δ(NH)) (proteins)
13	2847	2848	2847	−1	0	1	Symmetric stretching of the CH_2_ group due to mainly lipids, with little contribution from proteins, carbohydrates and nucleic acids
14	2915	2915	2915	0	0	0	CH_2_ asymmetric stretch: mainly lipids, with little contribution from proteins, carbohydrates, nucleic acids
15	2957	2956	2956	1	1	0	CH_3_ asymmetric stretch: mainly lipids
16	3284	3280	3283	4	1	−3	ʋ̵NH stretching of the peptide bond (–NHCO) of proteins and ʋ̵OH groups of water

**Table 3:** Pearson correlation (*p* < 0.05) test for Raman (a) and FTIR (b) spectra of control (*n* = 17, green dot); atypical hyperplasia (*n* = 12, black dot) and cancer (*n* = 16, red dot) tissues.

Raman Spectroscopy				FTIR Spectroscopy			
	Control *n* = 17	Atypical Hyperplasia *n* = 12	Cancer *n* = 16		Control *n* = 17	Atypical Hyperplasia *n* = 12	Cancer *n* = 16
**Control *n* = 17**	1.000	0.507 *	0.424	**Control *n* = 17**	1.000	0.966 *	0.966 *
**Atypical Hyperplasia** ***n* = 12**	0.507 *	1.000	0.747 *	**Atypical Hyperplasia** ***n* = 12**	0.966 *	1.000	0.950 *
**Cancer** ***n* = 16**	0.424	0.747 *	1.000	**Cancer** ***n* = 16**	0.966 *	0.950 *	1.000

* - Statistically significant, when *p* < 0.05.
